# Identifying Prokineticin2 as a Novel Immunomodulatory Factor in Diagnosis and Treatment of Sepsis*

**DOI:** 10.1097/CCM.0000000000005335

**Published:** 2021-09-27

**Authors:** Xiaoyan Yu, Jingyi Chen, Hong Tang, Qianqian Tu, Yue Li, Xi Yuan, Xuemei Zhang, Ju Cao, David Paul Molloy, Yibing Yin, Dapeng Chen, Zhixin Song, Pingyong Xu

**Affiliations:** 1 Department of Clinical Laboratory, Children’s Hospital of Chongqing Medical University, National Clinical Research Center for Child Health and Disorders, Ministry of Education Key Laboratory of Child Development and Disorders, Chongqing Key Laboratory of Child Infection and Immunity, Chongqing, China.; 2 Department of Critical Care Medicine, Department of Surgical Intensive Care Unit, the First Affiliated Hospital of Chongqing Medical University, Chongqing, China.; 3 Department of Biochemistry and Molecular Biology, Molecular Medicine and Cancer Research Center, Chongqing Medical University, Chongqing, China.; 4 Department of Laboratory Medicine, Key Laboratory of Diagnostic Medicine, Chongqing Medical University, Chongqing, China.; 5 Department of Laboratory Medicine, The First Affiliated Hospital of Chongqing Medical University, Chongqing, China.; 6 Department of Biochemistry and Molecular Biology, College of Basic Medical Sciences, ChongQing Medical University, Chongqing, China.; 7 Key Laboratory of RNA Biology, National Laboratory of Biomacromolecules, CAS Center for Excellence in Biomacromolecules, Institute of Biophysics, Chinese Academy of Sciences, Beijing, China.; 8 College of Life Sciences, University of Chinese Academy of Sciences, Beijing, China.

**Keywords:** immunoregulation, macrophage, prokineticin2, sepsis

## Abstract

Sepsis remains a highly lethal disease, whereas the precise reasons for death remain poorly understood. Prokineticin2 is a secreted protein that regulates diverse biological processes. Whether prokineticin2 is beneficial or deleterious to sepsis and the underlying mechanisms remain unknown.

**DESIGN:**

Prospective randomized animal investigation and in vitro studies.

**SETTING:**

Research laboratory at a medical university hospital.

**SUBJECTS:**

Prokineticin2 deficiency and wild-type C57BL/6 mice were used for in vivo studies; sepsis patients by Sepsis-3 definitions, patient controls, and healthy controls were used to obtain blood for in vitro studies.

**INTERVENTIONS:**

Prokineticin2 concentrations were measured and analyzed in human septic patients, patient controls, and healthy individuals. The effects of prokineticin2 on sepsis-related survival, bacterial burden, organ injury, and inflammation were assessed in an animal model of cecal ligation and puncture–induced polymicrobial sepsis. In vitro cell models were also used to study the role of prokineticin2 on antibacterial response of macrophages.

**MEASUREMENTS AND MAIN RESULTS:**

Prokineticin2 concentration is dramatically decreased in the patients with sepsis and septic shock compared with those of patient controls and healthy controls. Furthermore, the prokineticin2 concentration in these patients died of sepsis or septic shock is significantly lower than those survival patients with sepsis or septic shock, indicating the potential value of prokineticin2 in the diagnosis of sepsis and septic shock, as well as the potential value in predicting mortality in adult patients with sepsis and septic shock. In animal model, recombinant prokineticin2 administration protected against sepsis-related deaths in both heterozygous prokineticin2 deficient mice and wild-type mice and alleviated sepsis-induced multiple organ damage. In in vitro cell models, prokineticin2 enhanced the phagocytic and bactericidal functions of macrophage through signal transducers and activators of transcription 3 pathway which could be abolished by signal transducers and activators of transcription 3 inhibitors S3I-201. Depletion of macrophages reversed prokineticin2-mediated protection against polymicrobial sepsis.

**CONCLUSIONS:**

This study elucidated a previously unrecognized role of prokineticin2 in clinical diagnosis and treatment of sepsis. The proof-of-concept study determined a central role of prokineticin2 in alleviating sepsis-induced death by regulation of macrophage function, which presents a new strategy for sepsis immunotherapy.

Sepsis is designated as a highly lethal condition within the worldwide human population (1, 2) and with a current infection rate estimated to be between 47 and 50 million people, culminates in the demise of 11 million individuals annually (3). Clinical biochemical research has inevitably involved challenging and extensive studies of sepsis pathogenesis and survival (4–7), yet reasons for high morbidity rates remain poorly understood. Clinical diagnosis of the disease and treatments thereof to improve patient survival have provided evidence-based practices, although many of these have been deemed ineffective (8, 9) as, for example, *Drotrecogin alfa* (Xigris, activated protein C) was withdrawn after a decade of application (10). In view of a recent World Health Organization resolution (11), novel treatment regimens for sepsis are now being rigorously pursued.

Recent applications of molecular diagnostics have identified several contenders as biomarkers of sepsis (12); however, many fall short of the stringent criteria for translation into clinical use. For instance, procalcitonin, one enthusiastic biomarker in diagnoses of general sepsis, was transpired to be somewhat subjective in precision for diagnosis of early-onset neonatal sepsis for its naturally higher physiologic abundance and inequivalence in specificity (13, 14). Furthermore, time-consuming blood culture, once considered to be a mainstay diagnostic criterion, has, in fact, been overshadowed owing to a high frequency of false-negative results.

High mortality rates in patients afflicted with sepsis that derives multiple organ failure, are considered to involve, among others, inflammatory disorders (15, 16), immune cell paralysis (17–20), coagulopathy (21–23), and neuromuscular damage (24, 25). These ultimately pertain to immune dysfunction (26, 27). Our previous work revealed that representative macrophages, alongside a variety of bioactive cytokines, are central to the occurrence and development of sepsis, although the molecular mechanism for this is not well understood (28–32).

In this present study, we noted a surprising change in abundance of prokineticin2 in septic patients. This protein, originally identified as a member of the prokineticin family in *Bauhinia variegata* (33), was deemed to be a gastrointestinal secretory polypeptide involved in the regulation of peristalsis (34). Subsequently, prokineticin2 was also found to be widely expressed in a diverse array of tissues ranging from the CNS (35) and nonsteroidogenic cells of the testes (36) to immune cells (37, 38). Signal transduction by prokineticin2 is mediated via two structurally homologous receptors PKRs (PKR1 and PKR2) (39). Prokineticin2 is associated with a variety of biological functions including neurodevelopment (35, 40), angiogenesis (38, 41), circadian rhythms (42), reproduction (43, 44), and inflammatory discomfort (45). Alongside these, prokineticin2 promotes chemotaxis and alternative A2 reactivity of astrocytes (46).

Despite the overwhelming evidence in support for the role of prokineticin2 in multiple diseases, a direct correlation between the presence of this protein and the occurrence/development of sepsis, tied to a concomitant regulation of innate immunity and inflammation during sepsis, remains unclear. Here, we observed a significant correlation between the prokineticin2 hyposecretion and sepsis progression compared with a healthy control group. In an animal model, recombinant prokineticin2 (rPK2) administration significantly improved the survival rate of septic mice and somewhat alleviated sepsis-induced multiple organ damage associated with macrophage function. We thereby present a previously unrecognized aspect for the regulation of sepsis pathogenesis using prokineticin2-induced immunotherapy of the disease.

## MATERIALS AND METHODS

Detailed methods are available in the **Online Supplement** (http://links.lww.com/CCM/G858).

### Study Population

Adult patients and pediatric patients who met the clinical criteria for sepsis-3 along with the consensus and criteria recommended by the Society of Critical Care Medicine and the European Society of Intensive Care Medicine (1) were enrolled at admission to the ICU of the First Affiliated Hospital of Chongqing Medical University and the Children’s Hospital of Chongqing Medical University, respectively. Patients with malignancy, organ transplantation, immunodeficiency disease, autoimmune diseases, pregnancy, and the use of immunosuppressive medication in the past 2 months were excluded from the study. Nineteen adult and 10 pediatric nonseptic patients (patients with severe pneumonia who meets the diagnostic criteria for pneumonia [47, 48] but not for sepsis [1], Sequential Organ Failure Assessment score < 2) were recruited as patient controls. Specimens were collected after the patient was admitted to the ICU with sepsis or septic shock and before medical intervention. The clinical data, including WBCs, C-reaction protein and procalcitonin, microbial culture results, the length of ICU stay, or mortality during the 28-day study period, were recorded. Healthy control samples were obtained from healthy donors. This protocol was approved by the Clinical Research Ethics Committee of Chongqing Medical University (Institutional Review Board of Children’s Hospital of Chongqing Medical University, File No: [2019] Ethical Review [Clinical Research] No. 23), and informed consent was obtained from all participants according to the Declaration of Helsinki.

### Animal Model

Six- to eight-week-old male wild-type (WT) C57BL/6 mice (18–22g) were purchased from Beijing HFK Bioscience Co., Ltd, and heterozygous PK2 deficient mice (PK2^±^) C57BL/6 mice were purchased from Cyagen Biosciences Inc. All mice were raised at SPF laboratory of Chongqing Medical University. The animal experiments were done in accordance with the Chongqing Medical University Institutional Animal Care and Use Committee’s guidelines. A polymicrobial sepsis model was induced by cecal ligation and puncture (CLP) as described in our previous studies (28–30). Briefly, the mice were anesthetized with 1.5% pentobarbital sodium (75 mg/kg body weight) intraperitoneally, and then about 1 cm incision was made in the midline abdomen after skin disinfection. The cecum was exposed, ligated at the end 2/3, and punctured with a 26-gauge needle (nonsevere CLP) or with a 21-gauge needle (severe CLP). The cecum was then placed back in the peritoneal cavity, and the incision was closed with surgical staples. Sham-operated (control) animals underwent identical laparotomy operation; the cecum was exposed but not ligated or punctured. One milliliter of saline was subcutaneously administrated for resuscitation. Buprenorphine (0.05 mg/kg body weight) was injected intraperitoneally every 6 hours after surgery for postoperative pain relief until 48 hours after surgery. A humane endpoint was used for the lethal CLP model.

### In Vivo Administration of Recombinant Proteins

For in vivo prokineticin2 treatment, each mouse was injected intraperitoneally with 100 ng of murine rPK2 (PeproTech, Rocky Hill, NJ) or phosphate-buffered saline (PBS) after surgery and then 50 ng per mice intraperitoneally daily as a maintenance dose.

### Statistical Analysis

The data are presented as mean ± sd. Comparisons between groups were tested by Mann-Whitney *U* test. Log-rank tests were performed for survival studies. All analyses were done with GraphPad Prism Version 9.0.0 (GraphPad Software, San Diego, CA). *p* values less than 0.05 were considered statistically significant.

## RESULTS

### Prokineticin2 Concentration Is Significantly Decreased in Patients With Severe Sepsis

**Figure 1.**
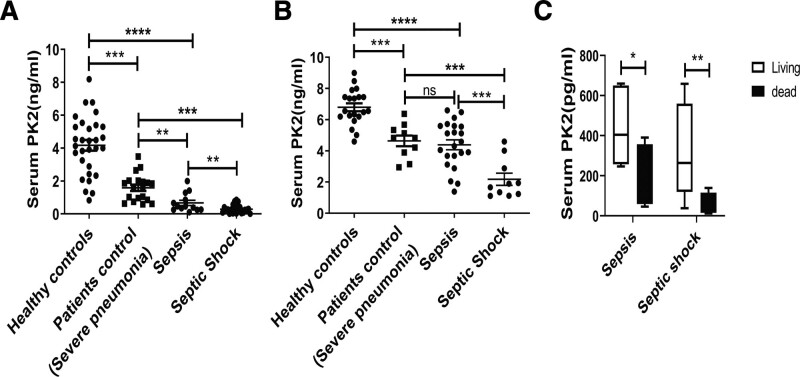
Serum prokineticin2 (PK2) levels were significantly decreased in patients with severe sepsis. **A**, PK2 concentrations were measured by enzyme-linked immunosorbent assay (ELISA) in serum samples collected from 47 adult patients with sepsis and septic shock, from 19 patients control with severe pneumonia, and from 30 healthy control subjects. **B**, PK2 concentration was measured by ELISA in serum samples collected from 31 pediatric patients with sepsis and septic shock, from 10 patients control with severe pneumonia, and from 20 healthy control subjects. **C**, The concentration of PK2 in serum were detected in adult patients who died of sepsis or septic shock and those who survived. *Horizontal bars* represent median values, and *dots* represent individual participants. *p < 0.05, **p < 0.01 compared between groups (denoted by *horizontal bracket*; Mann-Whitney *U* test).

As a start point in our analysis of the relationship between prokineticin2 and the progression of sepsis, blood samples from both adult and pediatric patients afflicted with sepsis and septic shock were assessed alongside patient controls and healthy control individuals (characteristics of patients with sepsis and controls are shown in **Supplemental Table 1** [http://links.lww.com/CCM/G859] and **Supplemental Table 2** [http://links.lww.com/CCM/G860]). Patients with severe pneumonia were used as a control for nonseptic infected patients. As expected, it was found that levels of prokineticin2 in both adult (*n* = 47) and pediatric (*n* = 31) patients with sepsis were significantly reduced with respect to healthy control subjects and patient control subjects. In addition, patients with septic shock had lower prokineticin2 than those with sepsis (Fig. 1, ***A*** and ***B***). Although the healthy control and patient groups were not well matched for age, we found no correlation between age and prokineticin2 serum concentration (data not shown) and do not think this difference explains the patterns seen. The above results suggest a potential role for prokineticin2 as a biomarker of sepsis and septic shock.

To further evaluate the role of prokineticin2 levels in predicting mortality in adult patients with sepsis and septic shock, we analyzed the relationship between prokineticin2 levels and mortality and found that patients with lower prokineticin2 levels had higher mortality. (Fig. 1*C*). This observation indicates that the concentration of prokineticin2 is inversely proportional to the severity of the disease. Overall, these data support that the notion of prokineticin2 in peripheral blood can be applied as a significant biomarker for progression and severity of the disease and predicting death in patients with sepsis and septic shock.

### Prokineticin2 Potentiation of Septic Mice Survival

**Figure 2.**
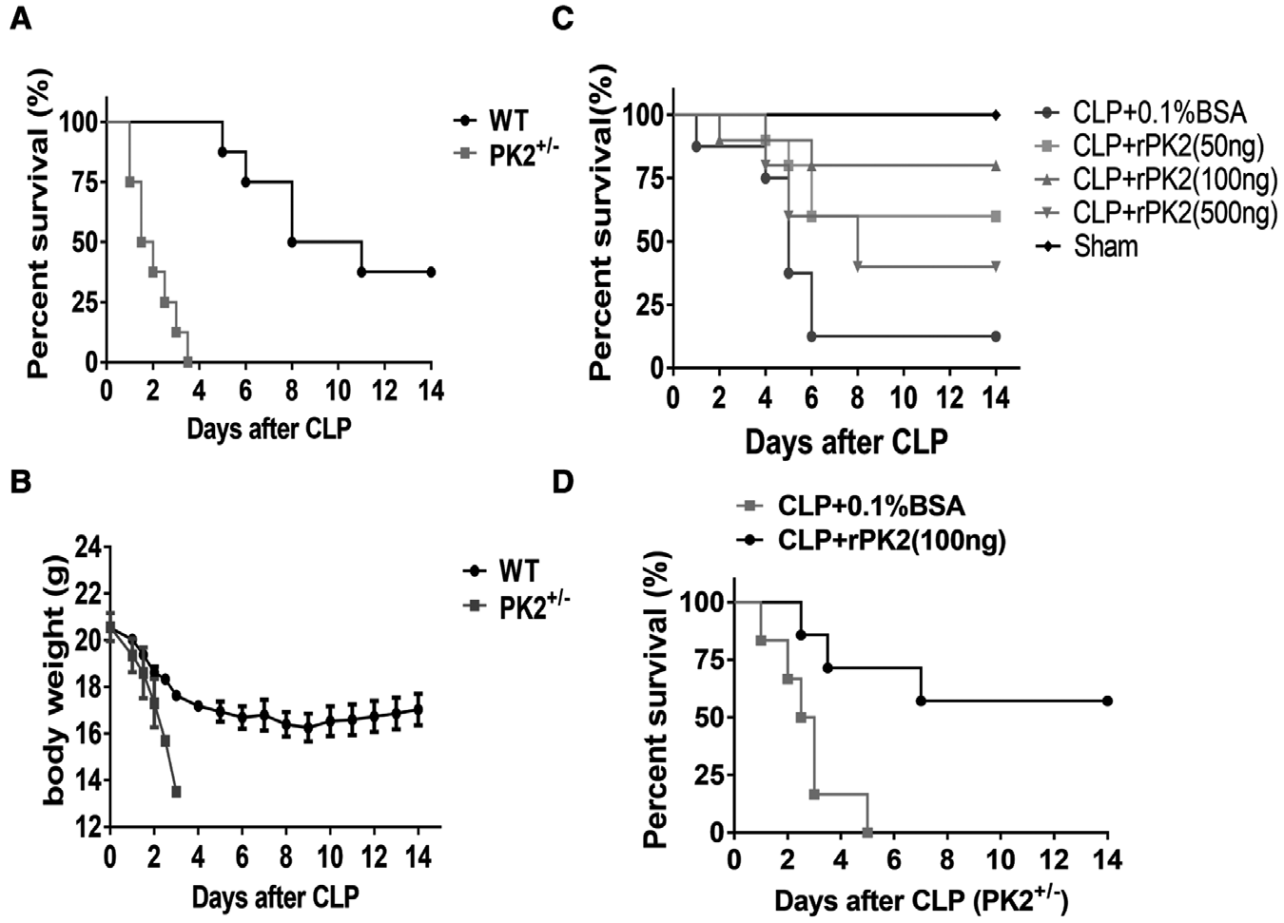
Administration of recombinant prokineticin2 (rPK2) protected mice from lethal experimental sepsis. **A** and **B**, Lethal sepsis model was induced by cecal ligation and puncture (CLP) with wild-type (WT) mice and heterozygous PK2 deficient mice (PK2^±^) mice; the survival and weight were then observed for 2 wk. Comparison of survival curves was by Log-rank (Mantel-Cox) test. **C**, C57BL/6 mice (18–22 g) underwent CLP to induce a lethal sepsis model (*n* = 8–12), followed by intraperitoneal injection of different doses of rPK2, with half the initial dose daily as a maintenance dose, and observed the survival for up to 2 wk. Comparison of survival curves was by Log-rank (Mantel-Cox) test. **D**, PK2^±^ mice (*n* = 5–8 per group) were subjected to lethal CLP, and then phosphate-buffered saline or rPK2 (100 ng/mouse) was administrated for each group. The survival rate was observed for 2 wk. Comparison of survival curves was by Log-rank (Mantel-Cox) test. BSA = bovine serum albumin.

To show a precise variation in patterns of prokineticin2 expression during the progression of sepsis, a CLP-induced sepsis mouse model was employed in which the levels of prokineticin2 were observed to increase mildly during onset of the disease and gradually diminished throughout the subsequent progression of sepsis (**Supplemental Fig. 1**, ***A*** and ***B***, http://links.lww.com/CCM/G861; **legend**, http://links.lww.com/CCM/G869). In view of the above, it was deemed essential to ascertain a more precise role of prokineticin2 in the onset and development of sepsis. Since it is difficult to obtain homozygous prokineticin2-deficient mice for their dysgeneses, we performed the survival rate experiment with prokineticin2 heterozygous (PK2^±^) mice and WT mice. The results showed that the survival rate of sepsis PK2^±^ mice (0%) was significantly lower than that of WT mice (37.5%) (Fig. 2*A*). Meanwhile, PK2^±^ mice lost weight faster than WT mice (Fig. 2*B*). Furthermore, the survival of septic mice after rPK2 administration was significantly improved in both WT mice and PK2^±^ mice (Fig. 2, ***C*** and ***D***). Moreover, significantly reduced bacteria loads were observed in samples of peritoneal lavage fluid (PLF), blood, and spleen tissues from within rPK2-treated septic WT mice and PK2^±^ mice compared with PBS control septic animals (**Supplemental Fig. 2**, ***A*** and ***B***, http://links.lww.com/CCM/G862; legend, http://links.lww.com/CCM/G869).

In addition, organ damage in sepsis progression observed through histologic examinations of hematoxylin eosin-stained tissues from PBS controls compared with rPK2-treated animals was performed (**Supplemental Fig. 3**, http://links.lww.com/CCM/G863; legend, http://links.lww.com/CCM/G869). Within PBS control animals, lung tissue injury was more prevalent as determined by severe hemorrhaging within, leukocyte infiltration of, exudation from, and destruction of alveolar wall tissue (Supplemental Fig. 3, http://links.lww.com/CCM/G863; legend, http://links.lww.com/CCM/G869). Furthermore, hepatic tissue damage within these animals was exacerbated in the formation of irregular hepatocyte arrays and severe vesicular degeneration accompanied by a significant infiltration of inflammatory cells compared with corresponding tissue from the rPK2-treated animals (Supplemental Fig. 3, http://links.lww.com/CCM/G863; legend, http://links.lww.com/CCM/G869). In spite of these, no morphologic changes were observed within spleen or renal tissues of the control group, although functional changes may exist. At the molecular level, biochemical markers indicative of tissue damage were partially reduced within rPK2-treated group (**Supplemental Fig. 2*C***, http://links.lww.com/CCM/G862; legend, http://links.lww.com/CCM/G869), and overall, these observations confirm a protective function of prokineticin2 during sepsis.

### The Protective Role of Prokineticin2 Is Macrophage Dependent

Increasingly, evidence supports the notion that macrophage involvement in sepsis could potentially alter the prognosis of the disease (28–31), although it is uncertain whether this bares similarities to their involvement in cancer and other inflammatory conditions (36, 49, 50). It also remains unclear whether rPK2 contributes to neutrophil and macrophage recruitment and activation during sepsis. In order to verify these processes, the WBC count in PLFs was obtained from improved Neubauer hemocytometry, and leucocyte morphology was observed by Wright’s staining in samples obtained from prokineticin2-treated and PBS control mice. No significant differences were observed in either WBC abundance or leucocyte morphology between the two groups (**Supplemental Fig. 4**, ***A*** and ***B***, http://links.lww.com/CCM/G864; legend, http://links.lww.com/CCM/G869). Thus, flow cytometry experiments were performed to ascertain whether any observable differences in the abundance of infiltrating leukocytes after CLP within PLF between the rPK2-treated and control groups could be observed. Once again, little or no discernable difference in either total cell count or proportions of different cell types was observed between the two groups (**Supplemental Fig. 4*C***, http://links.lww.com/CCM/G864; legend, http://links.lww.com/CCM/G869). These findings contradict previous observations which suggested prokineticin2 plays a role in neutrophil and macrophage chemotaxis (46, 51) despite the fact that phagocytic and bactericidal function of macrophages is significantly elevated in rPK2-treated animals (**Supplemental Fig. 4*D***, http://links.lww.com/CCM/G864 and **Supplemental Fig. 5**, http://links.lww.com/CCM/G865 [legend, http://links.lww.com/CCM/G869]).

**Figure 3.**
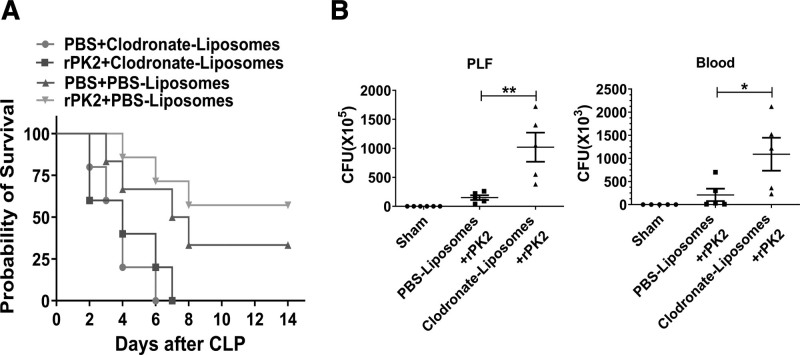
Prokineticin2 regulates macrophage function during sepsis. **A** and **B**, Forty-eight hours before the survival experiment and the bacterial load experiment, clodronate-encapsulated liposomes and phosphate-buffered saline (PBS)-encapsulated liposomes were intraperitoneally administered to exhaust the macrophages. C57BL/6 mice (18–22g, *n* = 5–8 per group) were subjected to lethal cecal ligation and puncture (CLP) surgery with a 26-gauge needle, followed by intraperitoneal injection of recombinant prokineticin2 (rPK2) (100 ng) or PBS, with half the initial dose daily as a maintenance dose, and observed the survival for up to 2 wk. Three independent experiments were performed. **p < 0.01, comparison of survival curves by Log-rank (Mantel-Cox) test. C57BL/6 mice (*n* = 5–8 per group) were subjected to sublethal CLP with a 26-gauge needle and then intraperitoneally injected with PBS or rPK2 (100 ng/mouse). Twenty-four/48 hours later, the number of bacterial colonies from blood, spleen, and peritoneal lavage fluid was counted after 24-hr culture with blood agar. *p < 0.05, **p < 0.01 when compared with control group (Mann-Whitney *U* test). CFU = colony-forming unit.

We further assessed the importance of macrophages in the survival of rPK2-treated septic mice after severe CLP by depletion of macrophage numbers in the presence of clodronate-encapsulated liposomes. In this, rPK2-treated septic mice survival was dramatically reduced when compared with PBS-encapsulated liposome-treated control mice (Fig. 3*A*). It is also noteworthy that bacterial clearances within the peritoneal cavity and in the blood were adversely affected owing to macrophage depletion within control animals (Fig. 3*B*). To determine whether prokineticin2 itself has a direct antimicrobial effect, the growth curves of the bacteria after the addition of rPK2 were also observed. However, no differences were observed in bacterial growth curves with different rPK2 concentrations (**Supplemental Fig. 6**, http://links.lww.com/CCM/G866; legend, http://links.lww.com/CCM/G869), indicating that prokineticin2 itself has no direct antibacterial effect. These findings clearly support the essential role ascribed to macrophages in prokineticin2-mediated protection against sepsis.

### Prokineticin2 Regulates Macrophage Function Through Prokineticin2-PKR1-STAT3 Pathway

Prokineticin2 treatment appears to be involved in sepsis regression and alleviates tissue damage presumably in a macrophage-mediated prokineticin2-dependent fashion. In addition, it has previously been suggested that two structural isoforms of PKRs (PKR1 and PKR2) mediate prokineticin2 signaling. Prokineticin2 can bind to and activate both receptors resulting in different biological functions (52). It was, therefore, deemed essential to investigate further the prokineticin2 signaling pathway within macrophages, and three additional experiments were performed.

First, prokineticin2 activity in enhancing the phagocytic and bactericidal function of macrophages was indirectly assessed by monitoring the levels of macrophage receptor with collagenous structure (MARCO), a scavenger receptor of macrophages involved in phagocytosis, and inducible nitric oxide synthase (iNOS), an enzyme involved in bactericidal activity within rPK2-treated and untreated control macrophages. Little or no significant difference in the expressions of either MARCO or iNOS could be discerned between rPK2-treated and PBS control macrophages (**Supplemental Fig. 7, *A*** and ***B***, http://links.lww.com/CCM/G867; legend, http://links.lww.com/CCM/G869). In addition, rPK2 treatment of macrophages did not significantly alter secretion of common inflammatory cytokines, including interleukin (IL)–6, tumor necrosis factor-α, IL-10, and IL-17A (**Supplemental Fig. 7*C***, http://links.lww.com/CCM/G867; legend, http://links.lww.com/CCM/G869). In addition, rPK2 treatment of macrophages did not significantly alter secretion of common inflammatory cytokines, including interleukin (IL)–6, tumor necrosis factor-α, IL-10, and IL-17A (Supplemental Fig. 6*C*, http://links.lww.com/CCM/G866; legend, http://links.lww.com/CCM/G869).

**Figure 4.**
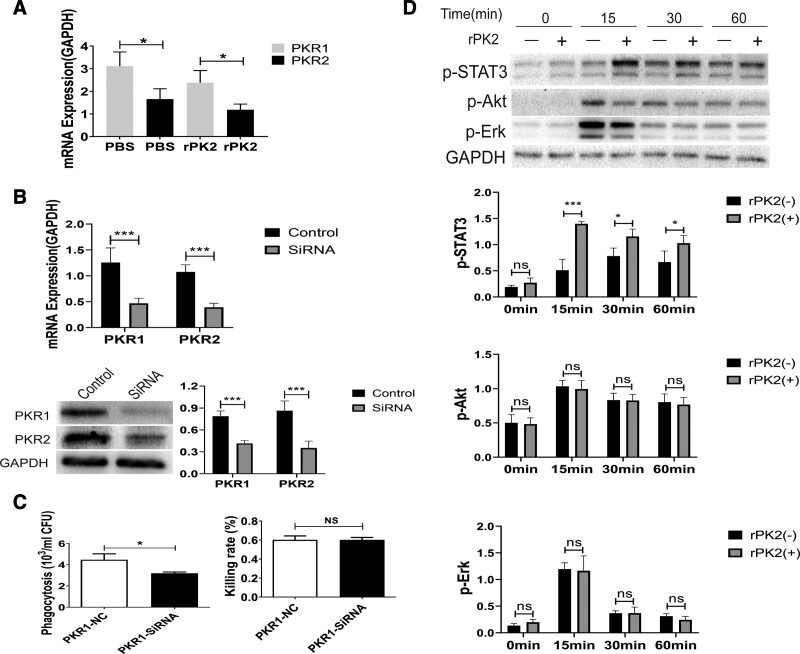
Prokineticin2 regulates macrophage function through prokineticin2-PKR1 pathway. **A**, The messenger RNA (mRNA) expression of prokineticin receptor 1 (PKR1) and prokineticin receptor 2 (PKR2) was determined by reverse transcriptase-polymerase chain reaction (PCR) (2^–ΔΔCt^). Data are expressed as mean values ± sd and were analyzed using the nonparametric Mann-Whitney *U* test. *p < 0.05, compared between two groups. **B**, The knockdown of the target PKRs were confirmed by quantitative PCR and WB. ***p < 0.001 when compared with control group (Mann-Whitney *U* test). **C**, The appropriate number of peritoneal macrophage (PMφ) were cultured in serum-free Dulbecco's modified eagle medium (DMEM) and transfected with the corresponding short interfering RNA (siRNA) reagents, and the transfection efficiency was detected. Then transfected cells were treated with phosphate-buffered saline (PBS) or recombinant prokineticin2 (rPK2) (10 ng/mL) for 12 hr and challenged with *Pseudomonas aeruginosa* for 30 min and then half of the cells plated to blood agar after a 10-fold series of dilution for phagocytosis. The other half of the cells were incubated another 90 min and then plated to blood agar after a 10-fold series of dilution for killing. *p < 0.05, **p < 0.01 when compared with control group (Mann-Whitney *U* test). **D**, rPK2 (10 ng/mL) and PBS-treated PMφ were collected and extracted protein for Western blot test. 2–ΔΔCt is a method for quantitative analysis of fluorescence data by quantitative reverse transcription-PCR. CFU = colony-forming unit, p-STAT3 = phosphorylated STAT3.

Second, quantitative polymerase chain reaction (qPCR) was employed to directly establish levels of expression from *PKR1*and *PKR2* genes in macrophages stimulated with heat-killed *Pseudomonas aeruginosa* (multiplicity of infection = 100). It was found that PKR1 expression is slightly higher than PKR2 (Fig. 4*A*). To further clarify the role of prokineticin2 binding with different PKRs in regulating macrophages, we conducted phagocytosis and bactericidal experiments after short interfering RNA (siRNA) knockdown of the PKR isoforms. The knockdown of the targets was confirmed by qPCR and WB (Fig. 4*B*). The results showed that after interfering with PKR1, phagocytosis activity of macrophages was significantly reduced, although bactericidal action was largely unaltered (Fig. 4*C*). Neither phagocytic nor bactericidal functions were significantly changed after knockdown of PKR2 (**Supplemental Fig. 7*D***, http://links.lww.com/CCM/G867; legend, http://links.lww.com/CCM/G869). These findings suggest that prokineticin2 regulates the bacterial clearance function of macrophages in a prokineticin2-PKR1–dependent manner.

Further studies of the signaling pathway revealed that rPK2 promoted STAT3 phosphorylation by 15 and 30 minutes in rPK2-treated macrophages compared with control, whereas other signaling pathways remained unaltered (Fig. 4*D*). Several studies have found that the STAT3 pathway is involved in the regulation of macrophage phenotype and function in a variety of diseases. Thus, phagocytosis experiments were performed after blocking STAT3 with the specific inhibitors S3I-20 and showed that prokineticin2 did not effectively enhance the phagocytic function of macrophages after STAT3 inhibition (**Supplemental Fig. 8**, http://links.lww.com/CCM/G868; legend, http://links.lww.com/CCM/G869). This finding suggests that prokineticin2 regulates the phagocytic function of macrophages in a prokineticin2-PKR1-STAT3–dependent manner.

## DISCUSSION

In the current study, a previously unrecognized role of prokineticin2 in regulating the host immune response during sepsis has been elucidated. This study determined a central role of prokineticin2 in alleviating sepsis-induced death by regulation of both macrophage function and inflammation response, which presents a new strategy for sepsis immunotherapy. This present study is of significance for both the diagnosis and treatment of sepsis. In terms of diagnosis, the insufficiency of prokineticin2 is closely related to deterioration in patient wellbeing, suggesting that continuous detection of prokineticin2 may be contributory to the diagnosis of sepsis/septic shock and the progress of recovery. Another important value of prokineticin2 we found is its ability to predict death in patients with sepsis and septic shock. The uniqueness of prokineticin2 as a novel biomarker in sepsis diagnostics lays in the fact that it is down-regulated during sepsis just similar to a negative acute phase reaction protein, whereas concentrations of existing markers appear to be elevated.

As treatment of sepsis, we demonstrate that prokineticin2 plays a protective role thereby improving survival rates in septic mice through enhancement of macrophage-mediated bacterial clearance. Animal experiments showed that the protective effect of prokineticin2 in sepsis was enhanced with the increase of the dosage in a certain range. So, replenishment of rPK2 may be a novel strategy for the treatment of sepsis and septic shock in patients with low prokineticin2 levels. These activities, therefore, circumvent an otherwise dire clinical prognosis for patients, where insufficient levels of prokineticin2 are a major hazard in sepsis progression, suggesting the value of prokineticin2 as a potential new drug. However, considering the concentration variation pattern of prokineticin2 in septic mouse models and the apparent biphasic response, it is worth noting that the safety dose range of prokineticin2 needs to be precisely determined in preclinical research through pharmaceutical studies, because excessive doses may have adverse effects as suggested by animal experiments. The other thing worth mentioning is that the CLP model may not completely mimic normal practice human fecal peritonitis as antibiotics were not used in the experiment, so more research on clinical and animal trials is needed.

Despite the significant protective effect of prokineticin2 during sepsis, the reason why prokineticin2 levels decrease during sepsis remains unclear. Previously, neutrophils and other WBCs were deemed to be important sources of prokineticin2 (49, 51). Moreover, sufferers of severe sepsis are also afflicted with immunosuppression, primarily as a consequence of a reduction in WBC numbers and immune cell paralysis, which must also surely result in significantly reduced levels of prokineticin2 expression. Moreover, a splice variant of prokineticin2, PK2L (a long form of prokineticin2, PK2β), which has a different in vivo distribution and function to prokineticin2, has recently been isolated (53). In addition, it remains unclear whether prokineticin2 levels physiologically change at 72 hours in neonates akin to the effects of other biomarkers; for example, procalcitonin which, thereby forced doctors to make additional adjustments when using it.

In this study, we found that prokineticin2 levels have potential value in predicting mortality in adult patients with sepsis and septic shock. However, this analysis was not performed in children because only two deaths in children with sepsis and septic shock were included in our data, which made statistical analysis difficult.

In the current study, we provided direct evidence that prokineticin2 enhances the phagocytic bactericidal function of macrophages. The regulation of prokineticin2 on macrophages provides us with a new understanding of the innate immune regulation of sepsis, which may help us to further reveal the pathogenic mechanisms of sepsis. In consideration of the fact that sepsis is a life-threatening, multiple organ dysfunctional condition as a consequence of impaired host response to infection and given that the receptor for prokineticin2 is widely expressed in other tissues (52), it is plausible that prokineticin2 also directly acts within other tissues to alleviate their dysfunction and thus improve survival. This notion also forms a basis for further study.

In a word, our study elaborated a previously unrecognized role of prokineticin2 in regulating the host immune response during sepsis, which provides new potential strategies for the diagnosis and immunotherapy of sepsis and septic shock.

## ACKNOWLEDGMENTS

We thank Lubei Rao (Department of Clinical Laboratory, Children’s Hospital of Chongqing Medical University) and Hongdong Li (Department of Neonatology, Children’s Hospital of Chongqing Medical University) for clinical samples collection.

## Supplementary Material


