# Supplementary material for: Identifying Prokineticin2 as a Novel Immunomodulatory Factor in Diagnosis and Treatment of Sepsis*
Source: Crit Care Med. 2021 Sep 27;50(4):674–84. doi: 10.1097/CCM.0000000000005335 (PMC8923365; doi:10.1097/CCM.0000000000005335)
Supplement: Supplementary file 1 [file ccm-50-0674-s001.docx]

**Determination of bacterial colony forming units (CFUs)**

After 24 hours and 48 hours of modeling, peritoneal lavage fluid (PLF), blood and spleen were collected. 100μl of PLF was plated on blood-agar plates according to the serial dilutions of 10^4^,10^5^,10^6^. 100μl blood and spleen homogenate were plated on blood-agar plates according to the serial dilutions of 10^2^,10^3^,10^4^, and the colony forming unit (CFU) counts were then determined after 24-hour culture.

**Tissue Histology**

Lungs, livers, spleens and kidneys were harvested after 24h and 48h of modeling and fixed in 4% paraformaldehyde (Shanghai Pharmaceuticals Holding Co.,ltd.). The paraffin-embedded tissue was cut into 5μm, and stained with hematoxylin-eosin (Sigma), and examined with microscopy (Nikon).

**Isolation of Murine Macrophages**

Mouse peritoneal macrophages (PMφs) were isolated from C57BL/6 mice as previously described ([1](#_ENREF_1)). In brief, eight-week-old male C57BL/6J mice were intraperitoneally injected with 2 ml of 3.0% thioglycollate medium (Sigma-Aldrich). Three days later, cells were harvested by peritoneal lavage with 5 ml of cold 0.05% EDTA-PBS. Collected cells were centrifuged at 500g/min for 10 min at 4°C, and the cells were washed with cold PBS and centrifuged again. The cells were then suspended in Dulbecco’s modified Eagle’s medium (Gibco) supplemented with 10% FBS (Gibco) and 1× penicillin-streptomycin (Sigma-Aldrich) and cultured in disposable plastic cell culture plates.

**Phagocytosis assays and** **bacterial killing assays**

For phagocytosis assays, PMφs were isolated, cultured and treated with rPK2 (Peprotech, 10ng/ml) or PBS and then incubated with heat-inactived FITC (Sigma, 0.5mg/ml) - labeled *P.aeruginosa* at a multiplicity of infection (MOI) of 100 for 30 minutes at 37°C. Cells were then washed and stained with DAPI (1μg/ml, Sigma-Aldrich) and TRITC-Phalloidin (Solarbio, 50nM), followed by visualization using fluorescence microscope (Nikon ECLIPSE 80i). The phagocytic rate (as determined by overlay of green bacteria) was quantified by an independent researcher from 300 counted cells per well and is expressed as percentage of cells that contain bacteria as described in our previous study ([2](#_ENREF_2)). The phagocytic index was quantified by counting the number of bacteria per 100 cells.

For bacterial killing assays, PMφs were infected with *P.aeruginosa* (MOI=100) at 37 °C for 30min, and they were washed with sterile PBS buffer containing tobramycin (100µg/ml) to remove extracellular bacteria and were lysed with Triton X-100 (Sigma, 0.2%). Live intracellular bacteria were quantified by culture of lysates for determination of bacterial uptake (t=30min) and intracellular killing (t=2h). Killing was calculated from the percentage of colonies present at t=2h as compared to t=30min, as follows: 1− [No. of CFUs t = 2h / No. of CFUs t = 30min].

**Determination of cytokine and chemokine**

The concentrations of Human Prokineticin2 (PK2) (Mybiosource), Mouse PK2 (CUSABIO), TNF-α (Biolegend), IL-6 (Biolegend), IL-10 (Biolegend) and IL-17A (Biolegend) were determined with commercially available ELISA kits according to the manufacturer’s instructions.

**Flow cytometry**

Mouse peritoneal cells were collected and stained with monoclonal antibodies against CD16/32 (eBioscience), CD11b(APC, eBioscience), Ly6G(FITC, BD Pharmingen), F4/80(PE, eBioscience) and correlative IgG Isotype Control. Flow cytometry (FCM) was then performed. At least 10^4^ cells were collected with a FACScan flow cytometer (BD) and analyzed with FCM Express software.

**Serum biochemistry**

Mouse blood was obtained 24 and 48 hours after modeling, and then serum was obtained after centrifugation. Serum biochemical markers including alanine aminotransferase (ALT), aspartate aminotransferase (AST), lactic dehydrogenase (LDH) and creatinine (Cr) were determined with commercial available kits (Sigma-Aldrich), using a Hitachi analyzer (Boehringer Mannheim) according to the manufacturers’ instructions.

**SiRNA interference PKR1/2**

PMφs were isolated and plated in a 6-well cell culture plate (1×10^6^/well) at 37℃ with 5% CO_2_. PMφs were then transfected with transfection complex including siRNA (50nM) and LipofectamineTM 2000 (diluted with DMEM), and cultured at 37℃ with 5% CO_2_ for 24-48h (replace fresh medium containing 1% FBS after transfection for 6h) to detect silence efficiency by RT-PCR and Western blot. Antibodies: anti-PKR1(NOVUS，NBP2-15201), anti-PKR2 (ORIGENE，TA321936).

SiRNA sequence (5’-3’) (Chongqing lebos biology):

si-PKR1: GAUCAGCAGUUCUACUACA tt; UGUAGUAGAACUGCUGAUC tt.

si-PKR2: GACCAUGUCUCCUUAAACU tt; AGUUUAAGGAGACAUGGUC tt.

**Western blot**

PMφs were isolated and plated in a 6-well cell culture plate (1×10^6^/well) at 37℃ with 5% CO_2_. PMφs were treated with rPK2 (10ng/ml, Peprotech) or PBS for 24h, and then challenged with heat-inactived *P.aeruginosa* at a multiplicity of infection (MOI) of 100 for different time point. Total cell proteins were extracted for Western blot analysis with primary antibodies against p-STAT3, p-Erk, p-Akt, GAPDH and HRP-linked IgG secondary antibody. All antibodies are purchased from Cell Signaling Technology (CST).

**In vivo microphage depletion**

The clodronate-encapsulated liposomes and PBS-encapsulated liposomes (Formumax Scientific Inc) were prepared as described in instructions. Six to eight-week-old male C57BL/6 mice were injected i.p. with 200μl of clodronate-encapsulated liposomes to deplete macrophages or with 200μl of PBS-encapsulated liposomes as a control. The macrophage was mostly depleted after 48 hours.

**References**

1.Song Z, Zhang X, Zhang L, et al. Progranulin Plays a Central Role in Host Defense during Sepsis by Promoting Macrophage Recruitment. Am J Respir Crit Care Med. 2016;194(10):1219-32.

2.Song Z, Zhang J, Zhang X, et al. Interleukin 4 Deficiency Reverses Development of Secondary Pseudomonas aeruginosa Pneumonia During Sepsis-Associated Immunosuppression. J Infect Dis. 2015;211(10):1616-27.
