# Supplementary material for: Identifying Prokineticin2 as a Novel Immunomodulatory Factor in Diagnosis and Treatment of Sepsis*
Source: Crit Care Med. 2021 Sep 27;50(4):674–84. doi: 10.1097/CCM.0000000000005335 (PMC8923365; doi:10.1097/CCM.0000000000005335)
Supplement: Supplementary file 2 [file ccm-50-0674-s002.docx]

**Supplemental Table 1.** Characteristics of adult patients with sepsis and healthy controls

| Characteristics | Sepsis patients  (n=47) | Severe pneumonia  (n=19) | Healthy controls  (n=31) |
| --- | --- | --- | --- |
| Sex (male/female) | 32/15 | 11/8 | 17/14 |
| Age (years) | 58 (36-95) | 56.95 (28-89) | 50 (26-67) |
| WBC | 8.85 (5.18-40.86) | 9.39 (2.02-27.86) | 6 (5-9) |
| CRP | 43.67 (17.3-90) | 41.84 (5-120) | NA |
| PCT | 28.80 (0.72-200) | 12.64 (0.058-100) | NA |
| Infection site  (NO. of patients) |  | | |
| Respiratory | 3 | 19 | NA |
| Abdominal | 40 | 0 | NA |
| Vascular | 0 | 0 | NA |
| Urinary | 3 | 0 | NA |
| Others | 3 | 0 | NA |
| Bacteremia | 17 | 0 | NA |
| Isolates (NO. of patients) |  | | |
| Gram positive | 6 | 0 | NA |
| Gram negative | 9 | 3 | NA |
| Fungus | 2 |  | NA |
| Miscellaneous | 0 | 0 | NA |
| APACHE II score | 22.2(14-45) |  | NA |
| ICU stay | 8 (2-96) |  | NA |
| Died/survived | 26/23 | 3/16 | NA |

NOTE: Data are expressed as median unless otherwise indicated. WBC: white blood cells; CRP: C-reaction protein; PCT: procalcitonin; APACHE II: acute physiology and chronic health evaluation II; ICU: intensive care unit; NA: not applicable.
