# Supplementary material for: Identifying Prokineticin2 as a Novel Immunomodulatory Factor in Diagnosis and Treatment of Sepsis*
Source: Crit Care Med. 2021 Sep 27;50(4):674–84. doi: 10.1097/CCM.0000000000005335 (PMC8923365; doi:10.1097/CCM.0000000000005335)
Supplement: Supplementary file 3 [file ccm-50-0674-s003.docx]

**Supplemental Table 2.** Characteristics of pediatric patients with sepsis and healthy controls

| Characteristics | Sepsis patients  (n=31) | Severe pneumonia  (n=10) | Healthy controls  (n=20) |
| --- | --- | --- | --- |
| Sex (male/female) | 12/19 | 5/5 | 13/7 |
| Age (years/months) | 3.11 (0.58-10) | 3.19 (0.10-8) | 6.25 (3-14) |
| WBC | 12.9 (3.91-39.87) | 12.38 (6.34-29.08) | 7.11 (3.94-9.89) |
| CRP | 43.23 (8-156) | 25.6 (8-112) | NA |
| PCT | 6.49 (0.555-100) | 1.28 (0.082-6.05) | NA |
| Infection site  (NO. of patients) |  | | |
| Respiratory | 21 | 10 | NA |
| Abdominal | 7 | 0 | NA |
| Vascular | 3 | 0 | NA |
| Urinary | 0 | 0 | NA |
| Others | 1 | 0 | NA |
| Bacteremia | 0 | 0 | NA |
| Isolates (NO. of patients) |  | | |
| Gram positive | 0 | 1 | NA |
| Gram negative | 0 | 3 | NA |
| Fungus | 0 | 0 | NA |
| Miscellaneous | 0 | 0 | NA |
| ICU stay | 1.77 (0-16) |  | NA |

NOTE: Data are expressed as median unless otherwise indicated. WBC: white blood cells; CRP: C-reaction protein; PCT: procalcitonin; ICU: intensive care unit; NA: not applicable.
