# Supplementary figures and images for: Identifying Prokineticin2 as a Novel Immunomodulatory Factor in Diagnosis and Treatment of Sepsis*
Source: Crit Care Med. 2021 Sep 27;50(4):674–84. doi: 10.1097/CCM.0000000000005335 (PMC8923365; doi:10.1097/CCM.0000000000005335)

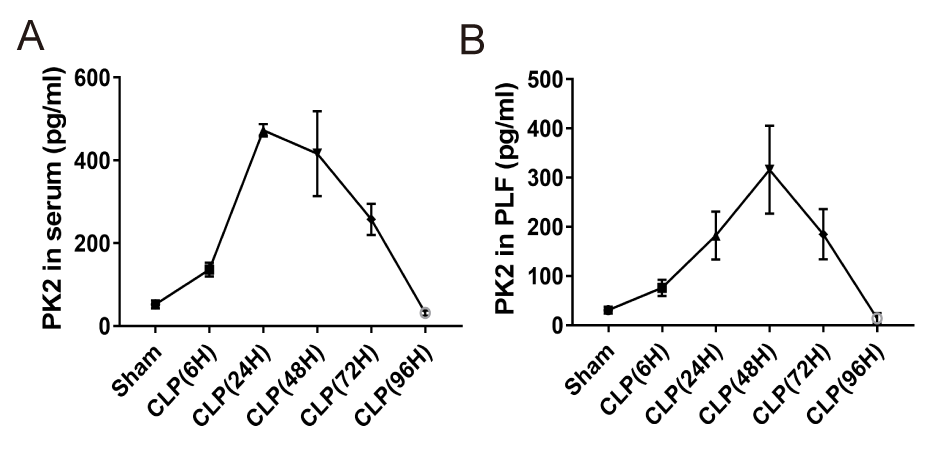

Supplement: Supplementary file 4 [file ccm-50-0674-s004.tif]

A

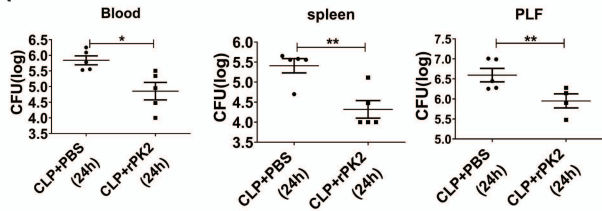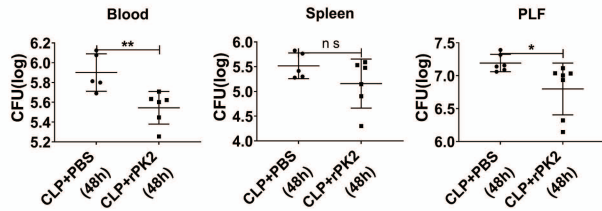

B

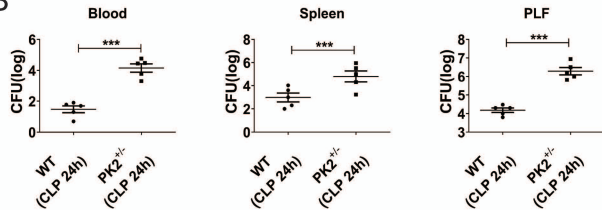

C

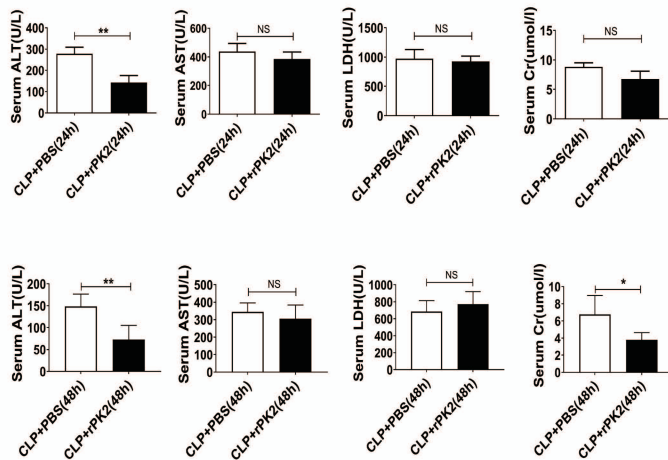

Supplement: Supplementary file 5 [file ccm-50-0674-s005.pdf]

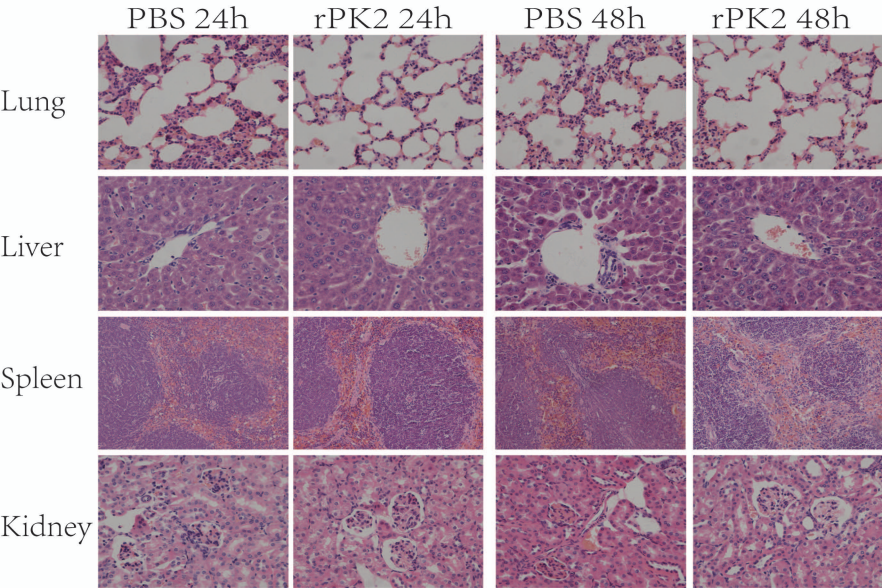

Supplement: Supplementary file 6 [file ccm-50-0674-s006.pdf]

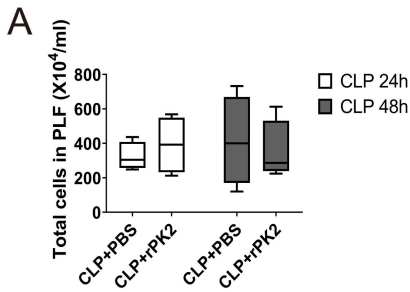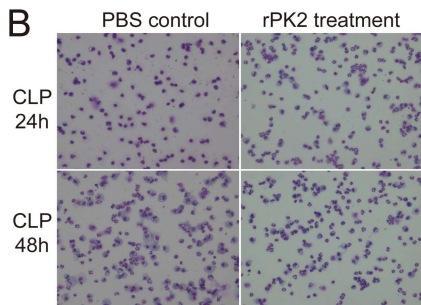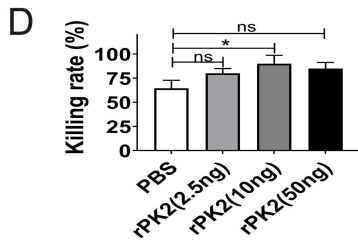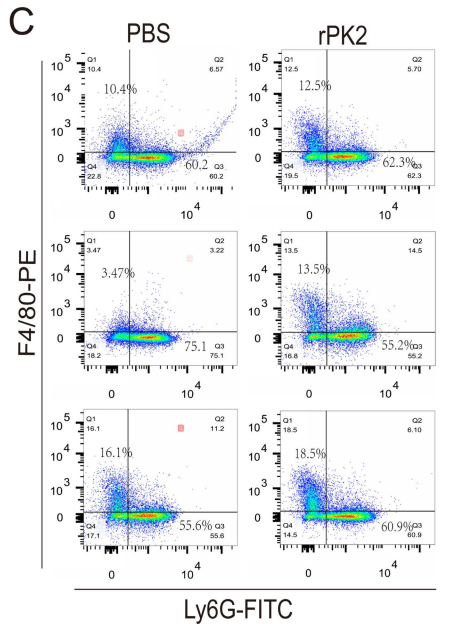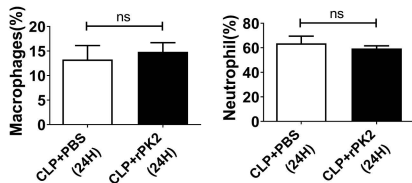

Supplement: Supplementary file 7 [file ccm-50-0674-s007.pdf]

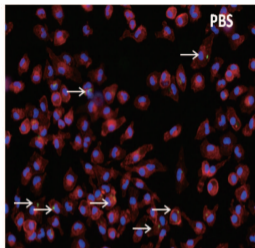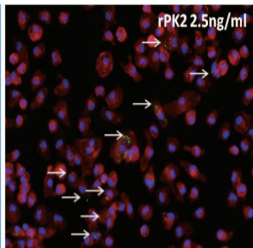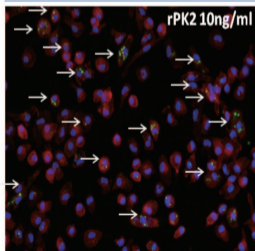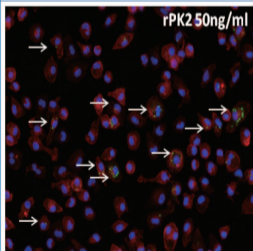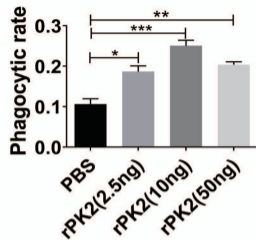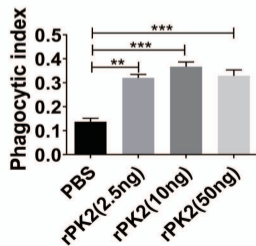

Supplement: Supplementary file 8 [file ccm-50-0674-s008.pdf]

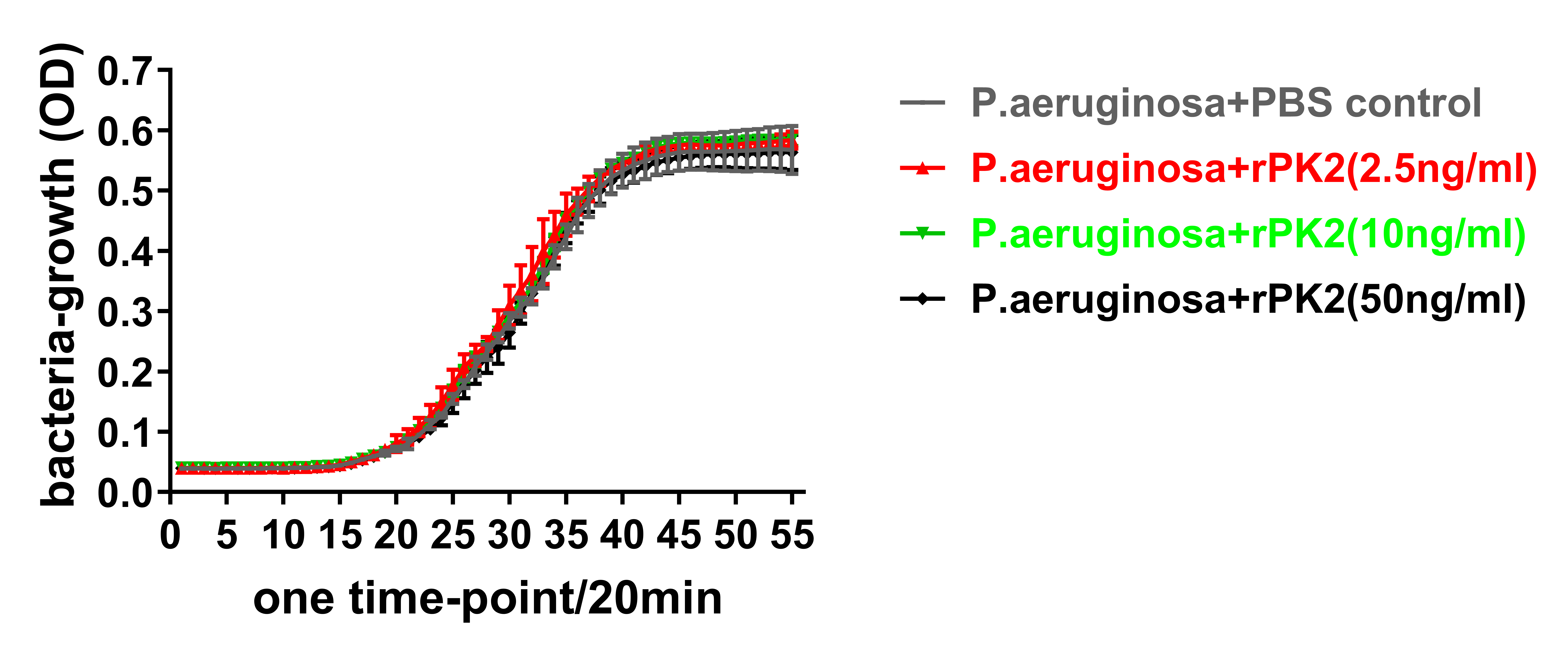

Supplement: Supplementary file 9 [file ccm-50-0674-s009.tif]

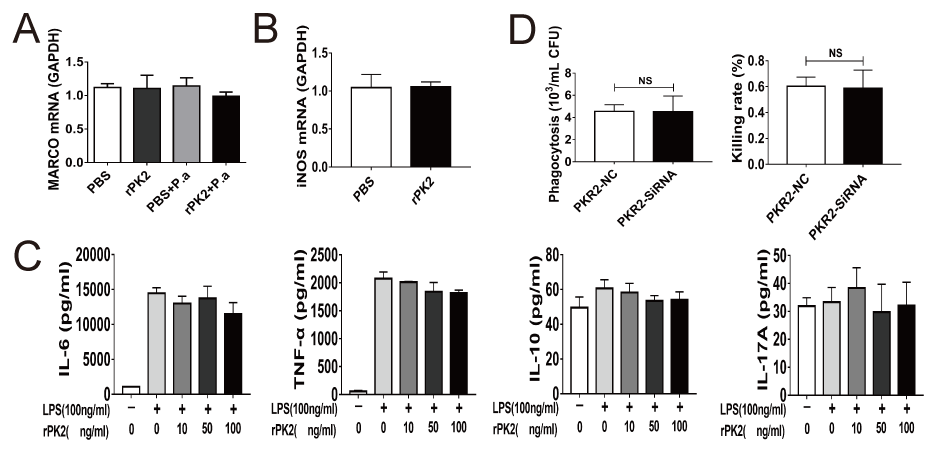

Supplement: Supplementary file 10 [file ccm-50-0674-s010.tif]

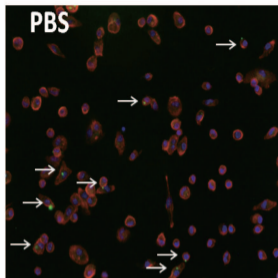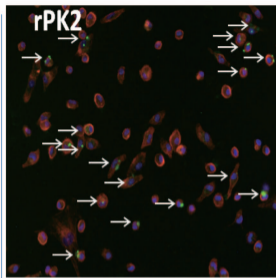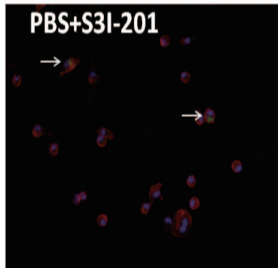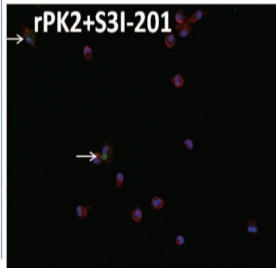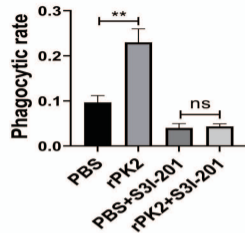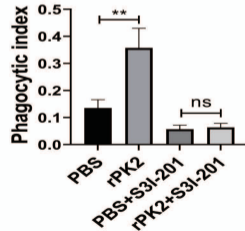

Supplement: Supplementary file 11 [file ccm-50-0674-s011.pdf]
