# Supplementary material for: Identifying Prokineticin2 as a Novel Immunomodulatory Factor in Diagnosis and Treatment of Sepsis*
Source: Crit Care Med. 2021 Sep 27;50(4):674–84. doi: 10.1097/CCM.0000000000005335 (PMC8923365; doi:10.1097/CCM.0000000000005335)
Supplement: Supplementary file 12 [file ccm-50-0674-s012.docx]

**Supplemental Figure 1.** PK2 concentration varies in a time-dependent manner in cecal ligation puncture (CLP)-induced sepsis model. C57BL/6 mice (n = 5-8 per group) were subjected to sham or sublethal CLP with a 26-gauge needle. PK2 concentration was measured by ELISA in serum samples (**A**)**,** and peritoneal lavage fluid (PLF) (**B**)**,** collected from sham and sepsis mice at different time points (n = 5–8). Data represent the mean ± SEM.

**Supplemental Figure 2.** Administration of rPK2 protected mice from lethal experimental sepsis. **A,** WT C57BL/6 mice (n = 5-8 per group) were subjected to sublethal CLP, and then PBS or rPK2(100ng/mouse) was administrated for each group. 24h/48h later, the number of bacterial colonies from blood, spleen and PLF was counted after 24h culture with blood agar. *P ＜ 0.05, **P ＜ 0.01 when compared with control group (Mann–Whitney U test). **B,** WT C57BL/6 or PK2^+/-^ mice were subjected to sublethal CLP, 24h later, the number of bacterial colonies from blood, spleen and PLF was counted after 24h culture with blood agar. ***P ＜ 0.001 when compared with control group (Mann–Whitney U test). **C,** C57BL/6 mice (n = 5-8 per group) were subjected to sublethal CLP, and then PBS or rPK2(100ng/mouse) was administrated for each group. Serological markers of organ injury including alanine aminotransferase (ALT), aspartate aminotransferase (AST), lactate dehydrogenase (LDH), and creatinine in rPK2 treated group or PBS control group (n = 5 per group) was detected by Roche Automatic Biochemical Analyzer. *P ＜ 0.05, **P ＜ 0.01 when compared with control group (Mann–Whitney U test).

**Supplemental Figure 3.** C57BL/6 mice (n = 5-8 per group) were subjected to sublethal CLP, and then PBS or rPK2(100ng/mouse) was administrated for each group. The examples of lung, liver, spleen, and kidney tissues from rPK2 treated group and PBS control group (n = 5 per group) was collected and stained with hematoxylin and eosin (HE) at different time points after CLP.

**Supplemental Figure 4.** PK2 regulates macrophage function during sepsis. C57BL/6 mice (n = 5-8 per group) were subjected to sublethal CLP with a 26-gauge needle and intraperitoneally administered PBS or rPK2(100ng). **A-B,** PLF was collected at the indicated time points after nonsevere CLP for WBC counting by improved Neubauer hemocytometer and Wright ‘ s staining. Data were expressed as mean values ± SD and were analyzed using the nonparametric Mann–Whitney U test. **C,** Flow cytometry (FCM) was performed to assess the ratio of macrophages and neutrophils in PLF (CLP 24h). The three images on the left show WBC in PLF of septic mice treated with PBS control and the three images on the right show WBC in PLF of septic mice treated with rPK2. The three images in each group represent three independent replicates. In each image, Region Q1 represents the proportion of macrophages and Region Q3 represents the proportion of neutrophils. **D,** PMφ were stimulated with different concentrations of rPK2 (2.5ng/ml, 10 ng/ml, 50ng/ml) or PBS for 12 hours and challenged with *P.a* for 30min and then half of the cells plated to blood agar after a 10-fold series of dilution for phagocytosis. The other half of the cells were incubated another 90 min and then plated to blood agar after a 10-fold series of dilution for killing. *P ＜ 0.05, **P ＜ 0.01 when compared with control group (Mann–Whitney U test).

**Supplemental Figure 5.** Mouse primary peritoneal macrophages (PMφs) were extracted and cultured, then stimulated with rPK2 or PBS for 12 hours and challenged with FITC-labeled *P.aeruginosa* (P.a) for 30 mins at 37℃. Cells were stained with DAPI (1μg/ml, Sigma-Aldrich) and TRITC-Phalloidin (50nM, Solarbio). Arrows indicate engulfed bacteria (as determined by overlay of green bacteria) by PMφ. Phagocytic and killing data are expressed as mean ± SD and were analyzed using the nonparametric Mann–Whitney U test. *P < 0.05, **P < 0.01, ***P < 0.001, compared with PMφ pretreated with rPK2.

**Supplemental Figure 6.** *P.aeruginosa* was cultured in Luria Bertani medium (LB medium) in 12-well cell culture plate, and the kinetics was monitored. The parameters are as follows: Instrument, Eon, BioTek Instruments USA. Total operation time, 18 hours. Time interval, 20 minutes. Total number of detections, 55. Wavelength, 600nm. Temperature, 37 ℃. The data were analyzed and plotted using GraphPad 9.0.0.

**Supplemental Figure 7.** PK2 regulates macrophage function through PK2-PKR1 pathway. **A-B,** the mRNA expression of MARCO and iNOS were determined by RT-PCR (2^-ΔΔCt^). Data are expressed as mean values ± SD and were analyzed using the nonparametric Mann–Whitney U test. *P < 0.05, compared between two groups. **C,** PMφs were isolated and plated in 24-well palte (1×10^5^/well), and then treated with rPK2 and LPS (100ng/ml). After 24h, the supernatant was collected for cytokine detection. Data were expressed as mean ± SD and were analyzed using the nonparametric Mann–Whitney U test. **D,** the appropriate number of PMφ were cultured in serum-free DMEM medium and transfected with the corresponding SiRNA reagents, and the transfection efficiency was detected. Then transfected cells were treated with PBS or rPK2, and phagocytosis and bactericidal experiments were performed as shown in **Supplemental Fig. 5**. Mann–Whitney U test.

**Supplemental Figure 8.** Phagocytic experiment was performed after the macrophages were treated with PBS, rPK2 (10ug/ml), PBS+S3I-201(100uM/ml) or rPK2+S3I-201. The detailed methods were referred to Fig.3A.
